# Enhanced light emission of quantum dot films by scattering of poly(zinc methacrylate) coating CdZnSeS/ZnS quantum dots and high refractive index BaTiO_3_ nanoparticles†

**DOI:** 10.1039/d0ra05389a

**Published:** 2020-08-28

**Authors:** Hongcheng Yang, Miao Zhou, Haodong Tang, Mingyu Sun, Pai Liu, Yizun Liu, Lixuan Chen, Dongze Li, Dan Wu, Junjie Hao, Bing Xu, Zhili Zhao, Zhenwei Ren, Siqi Jia, Kai Wang, Xiao Wei Sun

**Affiliations:** Guangdong University Key Laboratory for Advanced Quantum Dot Displays and Lighting, Shenzhen Key Laboratory for Advanced Quantum Dot Displays and Lighting, Department of Electrical and Electronic Engineering, Southern University of Science and Technology Shenzhen 518055 Guangdong China wangk@sustc.edu.cn sunxw@sustech.edu.cn; TCL China Star Optoelectronics Technology Co., Ltd. Shenzhen 518132 Guangdong China; School of Electronic and Computer Engineering, Peking University 518055 Shenzhen Guangdong China; Shenzhen Planck Innovation Technologies Co., Ltd. Shenzhen 518116 Guangdong China

## Abstract

Quantum dots (QDs) have received considerable attention in information displays owing to their high quantum yield, high colour purity and low-cost fabrication. However, light emission for ultra-thin QD films with low mass percentage of QDs still need to be improved because the blue light can directly transmit the films, leading to insufficient energy to excite the QDs. In this study, we report QD films based on a poly(zinc methacrylate) coating with alloyed green-emitting CdZnSeS/ZnS quantum dots (QDs@PZnMA) together with high refractive-index BaTiO_3_ nanoparticles to enhance the scattering coefficient of the QD films. Results demonstrate a 7.5-fold increase in the absorption coefficient, 11.3-fold increase in the scattering coefficient, 8.5-fold increase in the optical density (OD) and 8.6-fold increase in the green-light emission of QD films, compared with films that have the same mass percentage of pristine QDs. This approach provides a promising strategy for developing QD optical films with high scattering and enhanced light emission for flexible displays.

## Abstract 2

We report QD films based on a poly(zinc methacrylate) coating with alloyed green-emitting CdZnSeS/ZnS quantum dots (QDs@PZnMA) together with high refractive-index BaTiO_3_ nanoparticles to enhance the scattering coefficient of the QD films.

## Graphical abstract

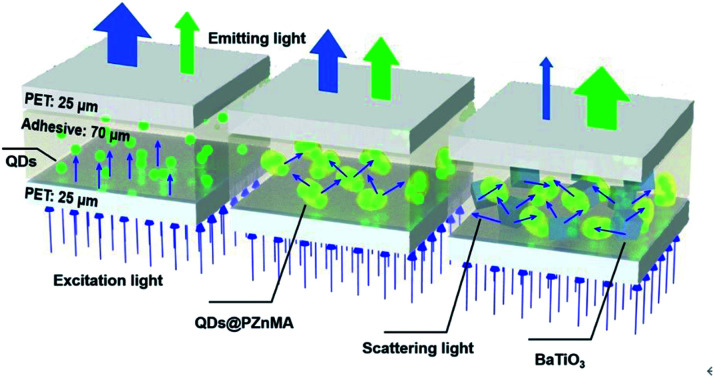

## 1. Introduction

Colloidal quantum dots (QDs), particularly cadmium-based QDs, have been intensively studied as light-conversion materials in lighting and display applications because of their narrow emission band, high colour purity, stability and tunable emission wavelength based on quantum confinement effects.^1–7^ Most QD televisions currently available in the market use QD films located on top of the light-guide plate. To display white, blue backlight, red- and green-emitting QDs are required. Moreover, the QDs act like phosphor for transforming blue into red or green light. However, the QD thin films used in display units are not as efficient as expected because of insufficient light absorption of excitation photons and internal reflections leading to reduced light emission of the QDs. Thus, many more QDs have to be introduced into QD films.^8^ This problem is particularly inevitable in ultra-thin display applications such as tablets and mobile phones, wherein QD films with a thickness of approximately 120 μm are required with high efficiency to convert the blue backlight by enhancing the QD light emission. Further, reducing the amount of cadmium-based QDs in optoelectronics is the ultimate goal for sustainability and drives us to make efforts to improve the light emission for QD films, particularly for ultra-thin QD films.

Scattering can alter the optical path and incident angle of the emitted light to increase internal reflection so that light emission of the optical film is enhanced through enhanced absorption for excitation photons.^9,10^ Li *et al.* showed that an eight-fold increase in the photoluminance (PL) emission of QD films can be obtained by attaining high reflectivity of the exciting light through photonic crystal structures on InGaN-based LED chips.^11^ Liu *et al.* applied micron-sized SiO_2_ particles as scattering particles to enhance the PL emission for QD films, but the increase of PL emission was not obvious because of the weak scattering properties of SiO_2_ particles with low refractive index (*n*) of approximately 1.4.^12,13^ Thus, it is necessary to further explore the effectiveness of high-refractive-index nanoparticles embedded in QD films to enhance the scattering properties.

In addition, unlike the traditional micron-sized phosphors possessing the scattering properties themselves,^14,15^ the blue light can easily leak through QD films owing to the ultra-small QDs, particularly for QD films with low concentrations and thicknesses of approximately 120 μm for flat-panel displays. Therefore, high-refractive-index nanoparticles, with their loss-less Mie scattering resonance, can be optimal for light modulation.^16,17^ Unlike high-refractive-index nanoparticles such as Ge, Si, Au and Ag nanoparticles exhibiting high absorption coefficient (*k*) to absorb the visible light,^18,19^ BaTiO_3_ nanoparticles exhibit a high refractive index (*n* ≈ 2.5), high transparency in the visible region and well-controlled size, morphology and crystallinity,^20^ which is expected to increase the utilisation of blue light and is promising for application in QD films.

**Scheme 1:**
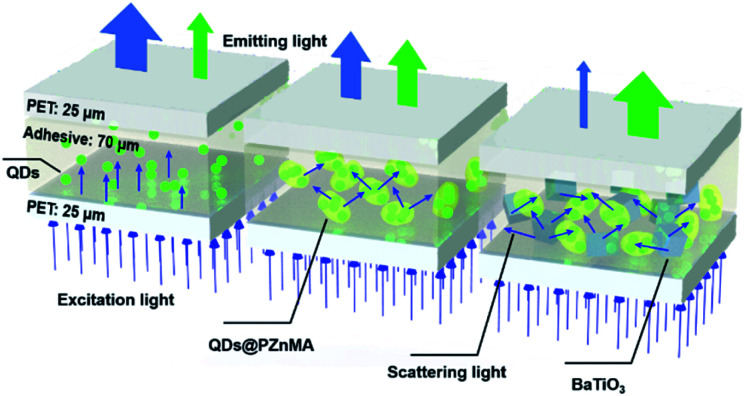
Illustration for light scattering within the QD films with the flexible poly(ethylene terephthalate) (PET) as protective layers: QD films made with pristine QDs (left), QDs@PZnMA (middle) and QDs@PZnMA with BaTiO_3_ as scattering particles (right).

In this study, to solve the above-mentioned problems of blue-light leakage and insufficient absorption for blue light by QDs, high-refractive-index BaTiO_3_ nanoparticles were selected as the scattering media to fabricate QD thin films, as shown in Scheme 1. In addition, the green-light conversion films were fabricated based on a poly(zinc methacrylate) coating with alloyed green-emitting CdZnSeS/ZnS QDs (QDs@PZnMA) because the S atoms on QDs can act as binding site for Zn atoms in zinc methacrylate, monoliths of which enhanced the scattering coefficient, optical density (OD) and the green-light emission, which was further enhanced using high-refractive-index BaTiO_3_ nanoparticles. The precise optical model of the QD films is discussed to determine the scattering properties in the QD films by combining the double integrating sphere (DIS) system with the inverse adding-doubling (IAD) algorithm. Results showed that the light-scattering function played an important role in enhancing the light emission of QD films.

## 2. Experimental

### 2.1 Reagents

Oleic acid (90%), 1-octadecence (ODE, 90%), cadmium oxide (CdO, 99.998%), trioctylphosphine (TOP, 97%), sulfur (S, 99.99%) and zinc acetate dihydrate (ZnAc_2_·2H_2_O, 99.99%) were purchased from Sigma. Selenium (Se, 99.999%) powder was bought from Alfa. Zinc methacrylate (95%), titanium butoxide (C_16_H_36_O_4_Ti, 99%), barium hydroxide octahydrate (Ba(OH)_2_·8H_2_O, 98%) and sodium hydroxide (NaOH, 97%) were purchased from Aladdin chemistry Co., Ltd. (Shanghai, China). UV-cured optical adhesive (acrylate type ingredient with isobornyl acrylate, C_13_H_20_O_2_) was acquired from POLOMO new material technology development co., LTD (Dongguan, China). All other organic solvents were purchased from Shanghai Lingfeng Chemical Reagent Co., Ltd.

### 2.2 Preparation of alloyed green-emitting CdZnSeS/ZnS quantum dots

Traditionally, the alloyed green-emitting QDs were synthesized by hot injection method. Briefly, the Se and S precursor solution were prepared by dissolving Se (1.4 mmol) and S (7.0 mmol) in TOP (4.5 mL) at 150 °C under nitrogen atmosphere. A mixture of CdO (0.4 mmol), Zn(Ac)_2_ (8.0 mmol), oleic acid (5.0 mL) and 1-octadecene (35.0 mL) was loaded in a 250 mL three-neck flask and heated to 250 °C to obtain a clear colorless solution under nitrogen protection. After the temperature reached to 300 °C, the above mixture Se/S-TOP precursor solution was rapidly injected to the flask under continuous stirring for 10 min. Finally, the synthesized green-emitting CdZnSeS/ZnS QDs were purified by centrifugation with the solvent/nonsolvent combination (hexane/ethanol) for three times.

### 2.3 Preparation of QDs@poly(zinc methacrylate) (PZnMA)

The QDs@PZnMA particles were synthesized by a solvothermal reaction. Simply, 1.0 mL QDs (50.0 mg mL^−1^ in ODE) and 200.0 mg zinc methacrylate were dispersed in 10.0 mL ODE solution additional with ultrasonic for 10 min. Then, the homogeneous solution was transferred into an autoclave with a Teflon liner and the reaction was proceeded up at 200 °C for 144 min. Finally, the particles were purified by centrifugation and washed with hexane for three times after the reaction cooling down to room temperature. To obtain the powder, the products could be acquired by freeze drying.

### 2.4 Preparation of BaTiO_3_ nanoparticles

The BaTiO_3_ nanoparticles were synthesized by a hydrothermal process. The precursor of C_16_H_36_O_4_Ti (215.8 mg) was dispersed in 10.0 mL ethanol, and Ba(OH)_2_·8H_2_O (200.0 mg) was dispersed in 10.0 mL ultra-pure water. Then the precursor mixed at the temperature of 80 °C to obtain the homogeneous solution with additional 1.0 mL NaOH (1.0 mol L^−1^). Then, the homogeneous solution was transferred into an autoclave with a Teflon liner and the reaction was proceeded up at 200 °C for 10 h. Subsequently, the products were purified by centrifugation, washed with ethanol and ultra-pure water for three times. Finally, the products of white powder could be obtained by freeze drying.

### 2.5 Fabrication of QD films

To evaluate the scattering and optical properties of QD films, four samples, with thickness of 120 μm, were fabricated with the low mass percentages of 0.66 wt% QDs, including three films with 5.00 wt% QDs@PZnMA monoliths (13.27 wt% QDs). Owing to the QDs@PZnMA with 13.27 wt% QDs in the PZnMA matrix, the 0.66 wt% QDs were in the films with 5.00 wt% QDs@PZnMA, including two films additional with 5.00 and 10.00 wt% BaTiO_3_ scattering particles. Therefore, all the QD films had the low mass percentage of 0.66 wt% QDs. The QDs/BaTiO_3_/QDs@PZnMA powders were mixed with the UV-cured optical adhesive with correlative mass percentage. After the vigorously stirring, the QD films were fabricated by coating machine with two layers of flexible poly(ethylene terephthalate) (PET) films (25 μm) as covering layers to make sandwich like structures. Finally, the QD films were solidified by UV irradiation for 30 seconds (365 nm, 80 mW cm^−2^).

### 2.6 Instruments

A PERSEE TU-1901 UV-Vis spectrophotometer was used to record UV-Vis absorption spectra of QDs. The PerkinElmer LAMBDA 950 was used to measure the wavelength dependence transmittance and absorbance of QD films. Photoluminescence quantum yield (QY) measurements were performed at with a C11347 (Hamamatsu) absolute QY measurement system. Fourier transform infrared (FT-IR) spectra were performed using KBr tablets with IR Tracer-100 (Shimadzu). Powder X-ray diffraction (XRD) patterns were performed on a Bruker D8 Advance X-ray Diffractometer at 40 kV and 40 mA using Cu Kα radiation (*λ* = 1.5406 Å). Transmission Electron Microscopy (TEM) images were collected by a FEI F30 transmission electron microscope with an accelerating voltage 300 kV and a Gatan SC 200 CCD camera. The high-angle annular dark-field scanning transmission electron microscopy (HAADF-STEM) and energy dispersive X-ray (EDX) spectra were performed using a FEI Talos F200S microscope with an accelerating voltage of 200 kV. The luminance of QD films was measured by OHSP-350L luminance meter (Hangzhou HOPOO optoelectronics technology Co., Ltd). The precise optical model of QD films including optical density (OD), scattering coefficient (*μ*_s_) and absorption coefficient (*μ*_a_) were measured by double-integrator system (DIS) (Everfine, Hangzhou) additional with inverse adding-doubling (IAD) algorithm. The refractive indices were measured using a dual rotating-compensator Mueller matrix ellipsometer (Wuhan Eoptics Technology Co., Wuhan, China). The QD films were fabricated by FA202D coating machine (Fuan Enterprise Development Co., Ltd, Shanghai, China) and the thicknesses of the films were controlled by the coating speed. The optical output power of the blue emitting LED (AISU 2835) for photoaging was measured using an ATA-500 Spectral Radiation Analyzer (EVERFINE Corporation) with an integrating sphere. All optical measurements were performed under ambient conditions.

## 3. Results and discussion

### 3.1 Characterisation of CdZnSeS/ZnS QDs@poly(zinc methacrylate)

**Fig. 1:**
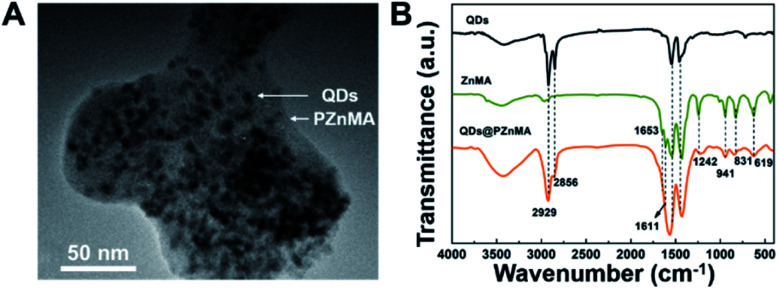
TEM image of QDs@PZnMA (A); Fourier transform infrared spectra of CdZnSeS/ZnS QDs, zinc methacrylate (ZnMA) and QDs@poly(zinc methacrylate) (B).

The morphology and optical characterisations of alloyed green-emitting CdZnSeS/ZnS QDs are shown in Fig. S1 and Table S1.† The as-synthesised QDs exhibited an average particle size of 10.5 nm. After zinc methacrylate co-polymerisation with the QDs to form monoliths of hundreds of nanometres, the QDs were randomly embedded in the PZnMA matrix with slight aggregation (Fig. 1A). Fourier transform infrared (FT-IR) spectra further confirmed the overall co-polymerised ZnMA with QDs. Referring to Fig. 1B, the 1546 cm^−1^ and 1419 cm^−1^ peaks were attributed to C=O and C–O of the acetate groups. After the solvothermal reaction with the QDs, the peak of the C=C bonds in the ZnMA monomer (1653–831 cm^−1^) evidently decreased, which demonstrates the formation of PZnMA.^21,22^ The large broad-bands from 1405 to 1598 cm^−1^ were coincident with those typically observed in acetate groups complexed with metallic zinc, corresponding to C=O stretching (1598 cm^−1^) and C–O stretching (from 1405 to 1459 cm^−1^).^23^ Interestingly, the existence of QDs in the reaction did not affect the zinc methacrylate polymerisation.

### 3.2 Characterisation of BaTiO_3_ nanoparticles

**Fig. 2:**
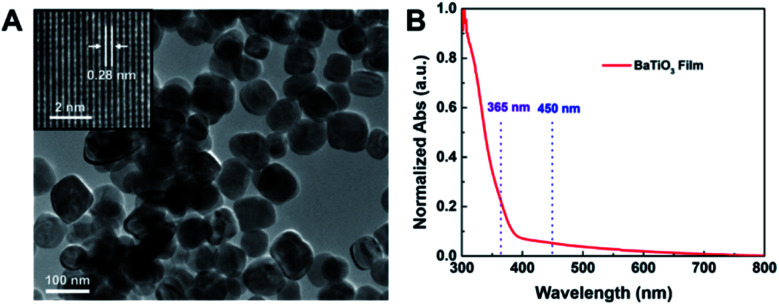
TEM image (A) and UV-Vis diffuse reflectance spectrum (B) of BaTiO_3_ nanoparticles.

BaTiO_3_ nanoparticles were synthesised through a hydrothermal process. As shown in Fig. 2A, the BaTiO_3_ nanoparticles, with a lattice spacing of 0.28 nm corresponding to the 〈110〉 direction, exhibited uniform morphology with an average particle size of approximately 84.2 nm (Fig. S3A†). The elemental and crystal structure characterisations are presented in the ESI (Figs. S3B–E†) and indicate a well-crystalised tetragonal phase. Furthermore, the UV-Vis diffuse reflectance spectrum (Fig. 2B) demonstrates that BaTiO_3_ nanoparticles exhibit weak absorption for visible light as well as the light corresponding to blue backlight and QD-emitted light in the process of multiple scattering. Therefore, the BaTiO_3_ nanoparticles can be applied to the QD films for scattering enhancement.

### 3.3 Scattering and optical properties of QD films

To investigate the optical properties of the QD films, four samples with a thickness of 120 μm, including two layers of 25 μm flexible poly(ethylene terephthalate) (PET) film as protective layers, were prepared, as showed in Scheme 1.

#### 3.3.1 Scattering properties of QD films

It was important to investigate the light-scattering behaviours within the QD films because the blue light passed through the films without being sufficiently absorbed by the QDs, which resulted in low light emission and an excessive amount of QDs in the films despite their high quantum efficiency. However, as shown in Fig. S4,† the light transmittance of the QD films does not precisely reveal the light-scattering properties because it only represents the diffuse reflectance spectra of the QD films. Therefore, to evaluate the scattering capabilities of nanoparticles and relevant QD films, a double integrating sphere (DIS) system (Fig. S5†) with an inverse adding-doubling (IAD) algorithm was employed to accurately analyse the scattering performance of these QD films. Based on our previous work,^24^ the scattering properties of QD films can be characterised through the scattering coefficient (*μ*_s_) and absorption coefficient (*μ*_a_), which were defined as the reciprocal of the average free path of the scattering (*I*_s_) and absorption events (*I*_a_), respectively. Therefore, a shorter *I*_s_ and larger *μ*_s_ would be expected for stronger scattering, whereas a stronger absorption coefficient (*μ*_a_) would be obtained for strong absorption of incident blue light of the QD films. Based on the measured reflectance, transmittance, collimated transmittance spectra and optical power, the scattering properties (*μ*_a_, *μ*_s_) of QD film samples were calculated using the IAD algorithm.

The absorption coefficient (*μ*_a_) of the QD films is shown in Table 1. Results show that the *μ*_a_ was only 0.33 mm^−1^ for pristine QDs and could be increased to 0.93 mm^−1^ after PZnMA coating. Importantly, the *μ*_a_ could be further enhanced to 2.19/2.48 mm^−1^ for samples (c/d), which is a 6.6/7.5-fold increase in *μ*_a_ compared with that of the contrast sample (a). By introducing the BaTiO_3_ nanoparticles, the optical path of light scattered in the QD films becomes longer, acquiring a higher probability of absorption because of the highly scattering media of BaTiO_3_ nanoparticles.

**Table 1:** The absorption coefficient (*μ*_a_) and scattering coefficient (*μ*_s_) of QD films

Labels	Samples/films	*μ*_a_ (mm^−1^)	*μ*_s_ (mm^−1^)
a	QDs	0.33	5.12
b	QDs@PZnMA	0.93	10.75
c	QDs@PZnMA + 5.00 wt% BaTiO_3_	2.19	36.32
d	QDs@PZnMA + 10.00 wt% BaTiO_3_	2.48	57.91

The scattering coefficient (*μ*_s_) indicates the scattering abilities of QD films for blue light at 450 nm, as shown in Table 1. For the contrast sample (a), the IAD algorithm calculation result for *μ*_s_ was only 5.12 mm^−1^ for the QDs film with 0.66 wt% QDs, signifying the weak scattering properties for this film. However, *μ*_s_ of the QD film could be increased to 10.57 mm^−1^ for sample (b) after PZnMA coating owing to the formation of monoliths with dimensions of hundreds of nanometres, which is a 2.1-fold increase compared with that of sample (a). Furthermore, the *μ*_s_ of QD films with QDs@PZnMA could be increased up to 36.32/57.91 mm^−1^ (for samples c/d) through doping with 5.00/10.00 wt% of BaTiO_3_ nanoparticles, which is a 7.1-/11.3-fold increase in *μ*_s_ compared with that of contrast sample (a), respectively, because of the high refractive index of BaTiO_3_ particles that facilitate excellent scattering properties (as shown in Fig. S6†).

#### 3.3.2 Optical properties for QD films

As shown in Table 2, the quantum yield (QY) of the films was increased from 71.2% to 78.3% after PZnMA coating of the QDs (Ex = 450 nm) because of the enhancement of the absorption of excitation photons of QDs through the scattering of QDs@PZnMA monoliths. However, BaTiO_3_ nanoparticles lead to a slight decrease in QY from 78.3% to 75.2/73.9% for QDs@PZnMA after doping with 5.00/10.00 wt% BaTiO_3_ nanoparticles, respectively. The slightly reduced QY is because of the slight absorption of BaTiO_3_ nanoparticles at 450 nm (Fig. 2B); however, despite this, the QY was still higher than that of films with pristine QDs. Therefore, this absorption peak would not hinder the application of the BaTiO_3_ scattering nanoparticle to the display and lighting fields in the visible light range.

**Table 2:** The quantum yield (QY, Ex = 450 nm), optical density (OD) and luminance of QD films^a^

Labels	QD films	QY (%)	OD	Luminance (cd m^−2^)
a	QDs	71.2	0.06	478.1
b	QDs@PZnMA	78.3	0.14	835.1
c	QDs@PZnMA + 5.00 wt% BaTiO_3_	75.2	0.36	1529.2
d	QDs@PZnMA + 10.00 wt% BaTiO_3_	73.9	0.51	1679.5

a The QY was measured by integrating sphere (Hamamatsu, C11347); optical density (OD) for 450 nm was performed by DIS system; the luminance was acquired by luminance meter on the cell phone blue emitting backlight (10 mW cm^−2^).

**Fig. 3:**
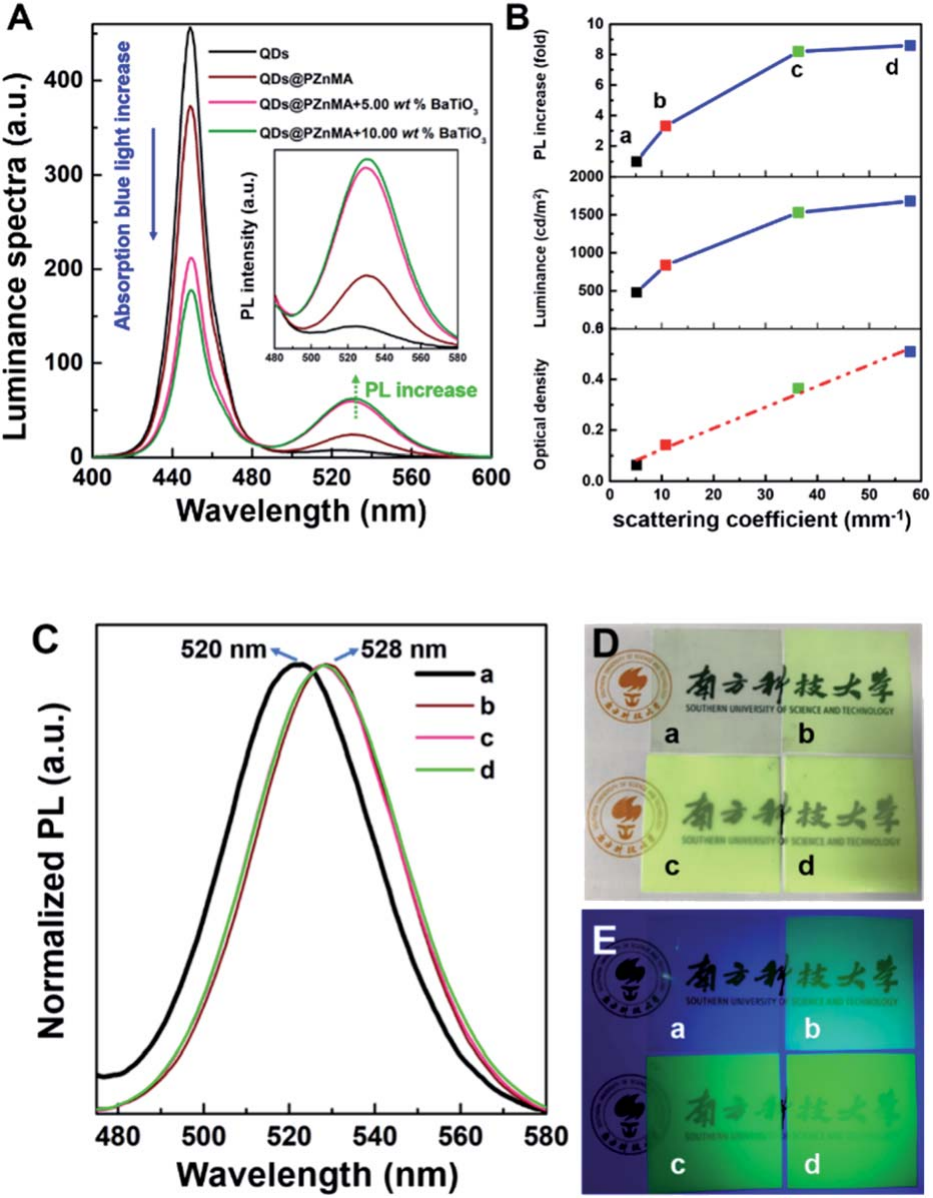
Luminance spectra acquired by luminance meter (A); the relationships between PL increase/luminance/optical density and scattering coefficient (B); PL spectra measured by integrating sphere (Hamamatsu, C11347) (C), photographs under white light (D) and UV light (E) for the films (5 cm × 5 cm) with QDs (a), QDs@PZnMA (b), QDs@PZnMA with 5.00 wt% BaTiO_3_ nanoparticles (c) and QDs@PZnMA with 10.00 wt% BaTiO_3_ nanoparticles (d), respectively.

The luminance spectra for the QD films are shown in Fig. 3A, and the relationship between the scattering coefficient and PL increase are shown in Fig. 3B. The green emission of the film with 0.66 wt% QDs, with a low scattering coefficient, was set as the reference (sample (a)). A 3.3-fold increase in green-light emission of sample (b) could be obtained through PZnMA coating the QDs for the enhancement of *μ*_s_ from 5.12 to 10.75 mm^−1^. More importantly, an 8.2-/8.6-fold increase in the green emission for samples (c/d) was observed for the QDs@PZnMA monoliths mixed with 5.00/10.00 wt% BaTiO_3_ nanoparticles compared with that of sample (a), respectively. Therefore, the BaTiO_3_ nanoparticles demonstrated significant capability in improving the green-light emission of these QD films.

The OD, acquired from the DIS system, indicated a specific assessment of the light transmittance of blue light of the QD films at 450 nm. As shown in Table 2, the contrast sample (a) with 0.66 wt% QDs exhibited a low OD of 0.06 at 450 nm, indicating that the blue light could be transmitted by the films efficiently because of the low concentration as well as the small particle size of the QDs. However, QDs@PZnMA monoliths sized hundreds of nanometres exhibited a significant decrease in the transmittance of blue light with an OD of 0.14 for sample (b). Furthermore, by introducing 5.00/10.00 wt% of BaTiO_3_ scattering nanoparticles, the OD of the QD films presented a significant increase to 0.36/0.51 for samples (c/d), respectively, representing a 6.0-/8.5-fold increase in the OD compared with that of the contrast sample (a). In addition, the relationship between the scattering coefficient and the OD is shown in Fig. 3B, which indicated that the increase of OD was positively related to the high scattering properties. Therefore, BaTiO_3_ nanoparticles and QDs@PZnMA monoliths play an important role in reducing the light transmittance to take full advantage of the blue light.

Based on the PL spectra shown in Fig. 3C, the PL peak of QDs@PZnMA showed a slight red shift from 520 to 528 nm, which was explained through the weaker quantum confinement effect after PZnMA coating the QDs owing to the mass increase.^25,26^ According to Table 2 and the relationship between scattering coefficient and the luminance shown in Fig. 3B, for the pristine QDs, this film exhibited a low luminance of 478.1 cd m^−2^ when excited by the blue emitting backlight of a cell phone. However, the luminance of the QDs@PZnMA film reached 835.1 cd m^−2^ under the same conditions. Moreover, the luminance was further enhanced to 1529.2/1679.5 cd m^−2^ for samples (c/d) with 5.00/10.00 wt% BaTiO_3_ nanoparticles, respectively, representing 1.8-/2.0-fold increases in luminance compared with the sample (b). Apparently, a major mechanism for the enhancement in luminance is to increase light emission through strong scattering. These strategies for the increase in luminance will significantly reduce the mass percentage of QDs in these films, reducing the heavy metal content and effectively increasing the light-conversion efficiency.

The appearances of the QD films under white light and UV light are shown in Fig. 3D and E, respectively. For samples (c and d), these films were extremely cloudy under white light when QDs@PZnMA was fabricated with BaTiO_3_ nanoparticles. Moreover, it was evident that the QD films containing BaTiO_3_ nanoparticles exhibited a higher brightness for samples (c and d) compared with that of the contrast sample (a) under UV light, indicating that the enhancement in scattering amplified the luminescence intensity to effectively attain high brightness in QD films.

## 4. Conclusions

In summary, we proposed high-luminance QD films with QDs coated with poly(zinc methacrylate) (QDs@PZnMA) and BaTiO_3_ nanoparticles as a scattering material. The coating of QDs was achieved through a solvothermal reaction to induce polymerisation, whereas the BaTiO_3_ nanoparticles were synthesised through a hydrothermal process. Owing to the enhancement of scattering in the QD films, a 7.5-fold increase in the absorption coefficient, 11.3-fold increase in the scattering coefficient, 8.5-fold increase in the optical density and an 8.6-fold increase in green-light emission were achieved compared with those of pristine QD films. These results represent remarkable progress in the enhancement of light emission and scattering for QD thin films fabricated with a low mass percentage of QDs. We provide reasonable guidelines for effective maximum light efficiency and reduce the power consumption in optoelectronics and flexible displays.

## Conflicts of interest

The authors declare no conflict of interest.

## Supplementary Material

RA-010-D0RA05389A-s001
