# Supplementary material for: Enhanced light emission of quantum dot films by scattering of poly(zinc methacrylate) coating CdZnSeS/ZnS quantum dots and high refractive index BaTiO3 nanoparticles
Source: RSC Adv. 2020 Aug 28;10(53):31705–10. doi: 10.1039/d0ra05389a (PMC9056395; doi:10.1039/d0ra05389a)
Supplement: RA-010-D0RA05389A-s001 [file RA-010-D0RA05389A-s001.pdf]

Electronic Supplementary Material (ESI) for RSC Advances.
This journal is © The Royal Society of Chemistry 2020

Supplementary Information

Enhanced Light Emission of Quantum Dot Films by Scattering of Poly Zinc Methacrylate Coating CdZnSeS/ZnS Quantum Dots and High Refractive Index $BaTiO_3$ Nanoparticles

Hongcheng Yang,[a,d] Miao Zhou,[b] Haodong Tang,[a] Mingyu Sun,[a] Pai Liu,[a] Yizun Liu,[d] Lixuan Chen,[a,b,c] Dongze Li,[b] Dan Wu,[a] Junjie Hao,[a,d] Bing Xu,[a,d] Zhili Zhao,[a] Zhenwei Ren,[a] Siqi Jia,[a] Kai Wang*[a] and Xiao Wei Sun*[a]

[a]Guangdong University Key Laboratory for Advanced Quantum Dot Displays and Lighting, Shenzhen Key Laboratory for Advanced Quantum Dot Displays and Lighting, Department of Electrical and Electronic Engineering, Southern University of Science and Technology, Shenzhen 518055, Guangdong, China.

[b]TCL China Star Optoelectronics Technology Co., Ltd., Shenzhen 518132, Guangdong, China.

[c]School of Electronic and Computer Engineering, Peking University 518055, Shenzhen, Guangdong, China.

[d]Shenzhen Planck Innovation Technologies Co., Ltd., Shenzhen 518116, Guangdong, China.

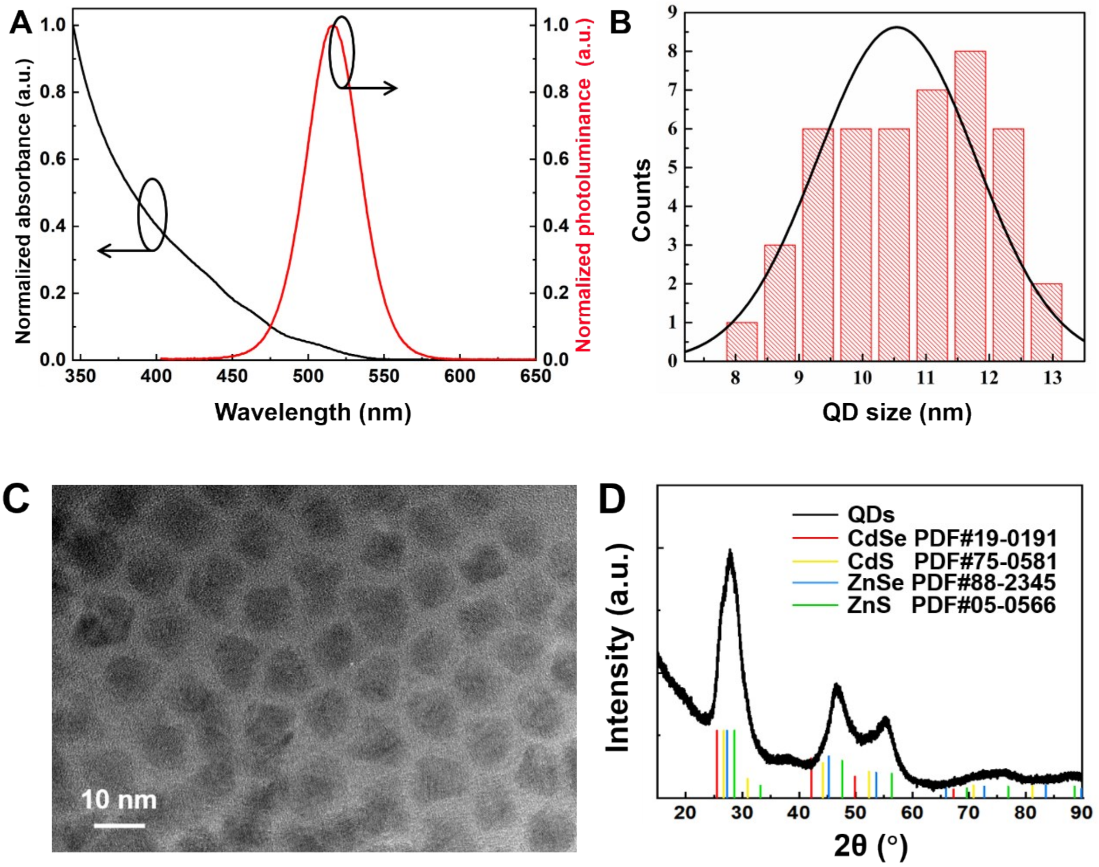

Fig. S1 Absorption, photoluminance spectrum (A) and size distribution (B) of CdZnSeS/ZnS QDs; TEM image of QDs (C); X-ray di□raction patterns of QDs, the vertical solid lines indicate zinc blende CdSe (red), CdS (yellow), ZnSe (blue) and ZnS (green) bulk reflections (D).

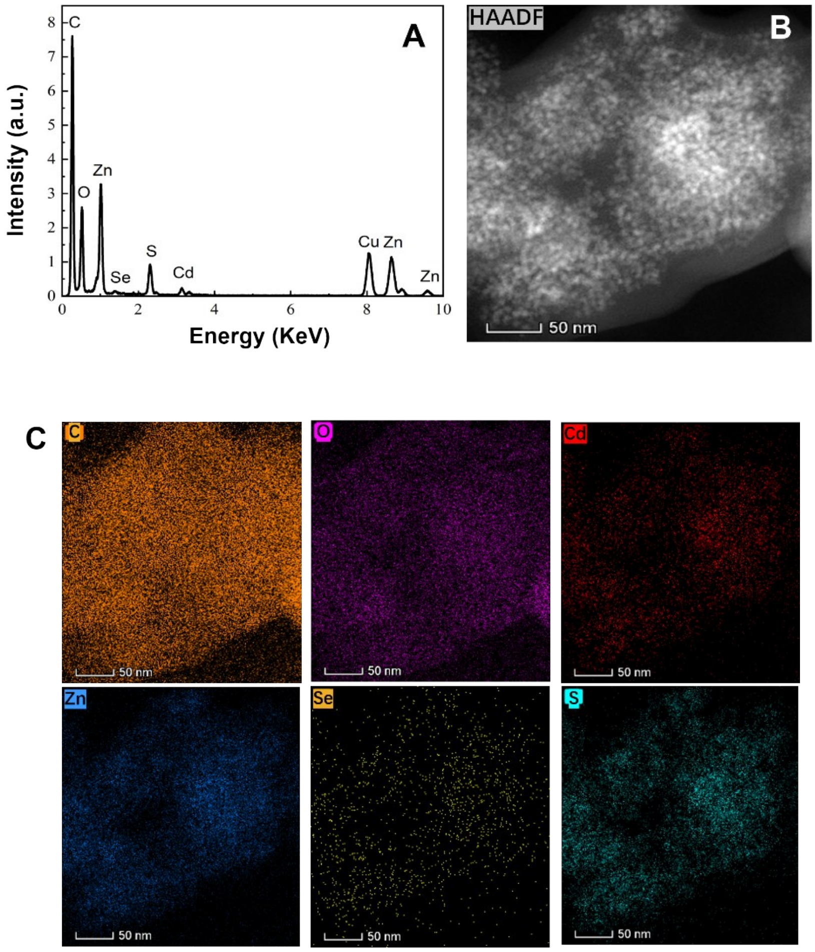

Fig. S2 EDX spectrum (A), HAADF-STEM image (B) and EDX mapping of C, O, Cd, Zn, Se, S elements (C) for QDs@PZnMA.

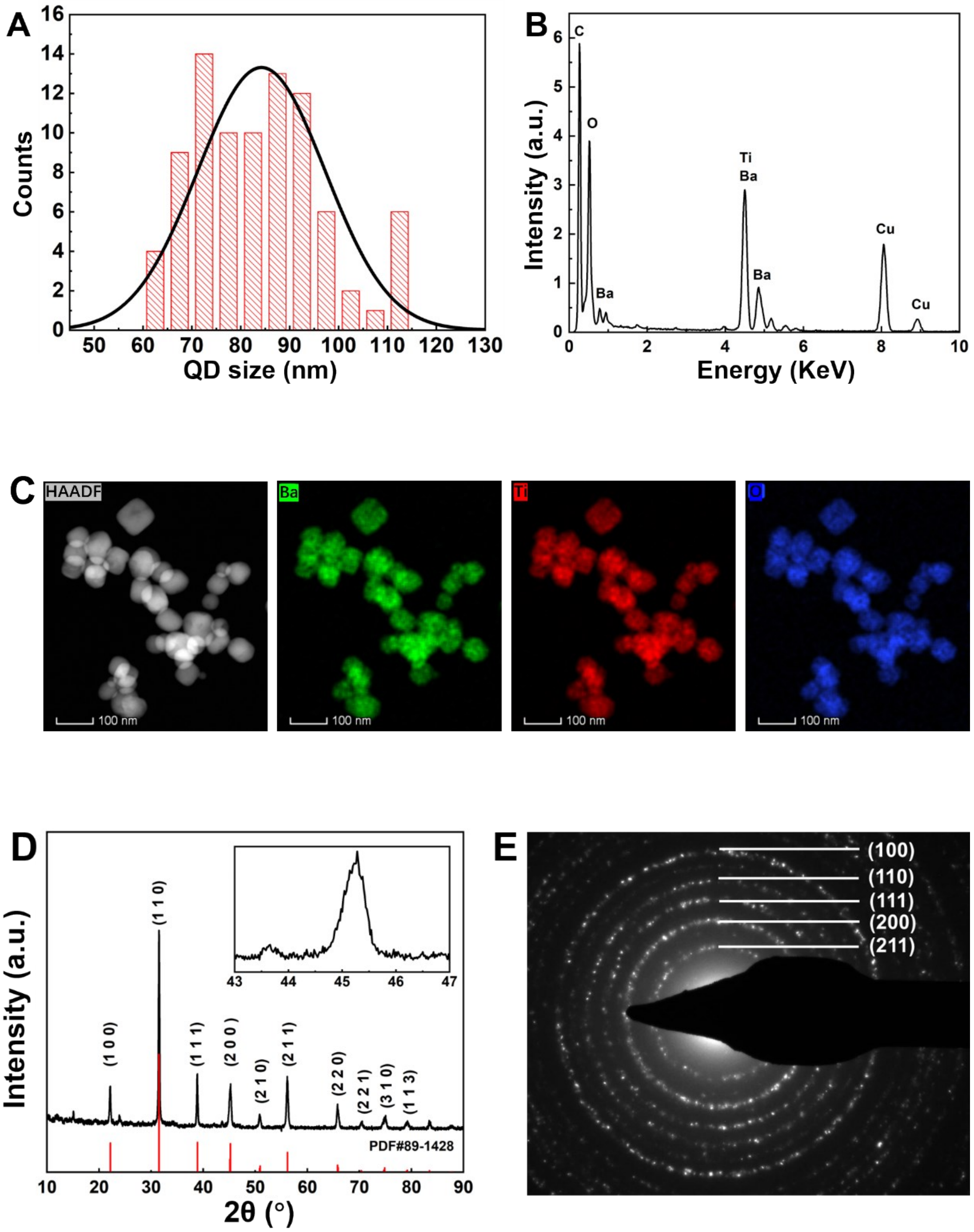

Fig. S3 The size distribution (A), EDX spectrum (B), HAADF-STEM image and EDS mapping (in STEM mode) of Ba, Ti and O elements (C) for $BaTiO_3$ nanoparticles; XRD pattern of $BaTiO_3$ particle (the bottom pattern is the standard XRD pattern of tetragonal phase PDF#89-1428) (D) and selected area electron diffraction of $BaTiO_3$ particle (E).

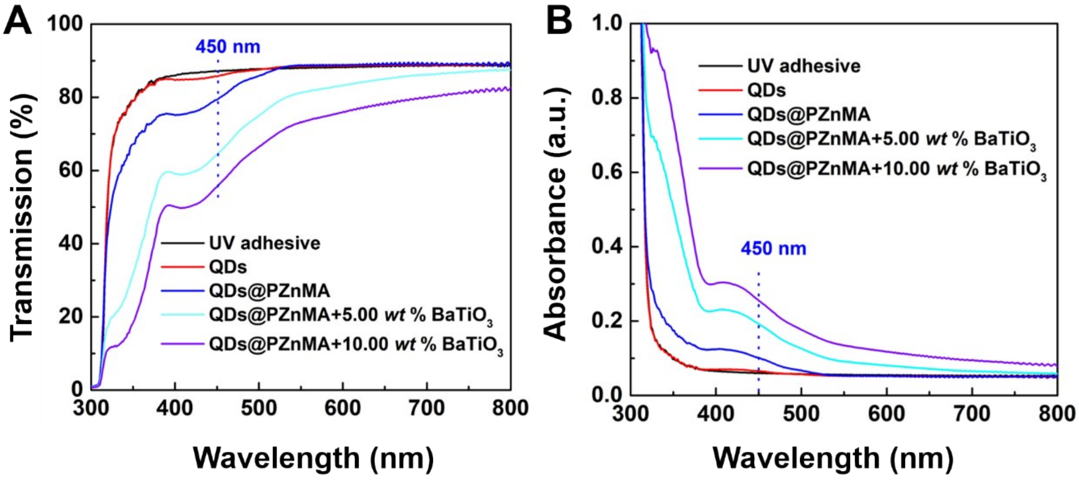

Fig. S4 The wavelength dependence of direct transmittance (A) and the corresponding absorbance (B) for QD films.

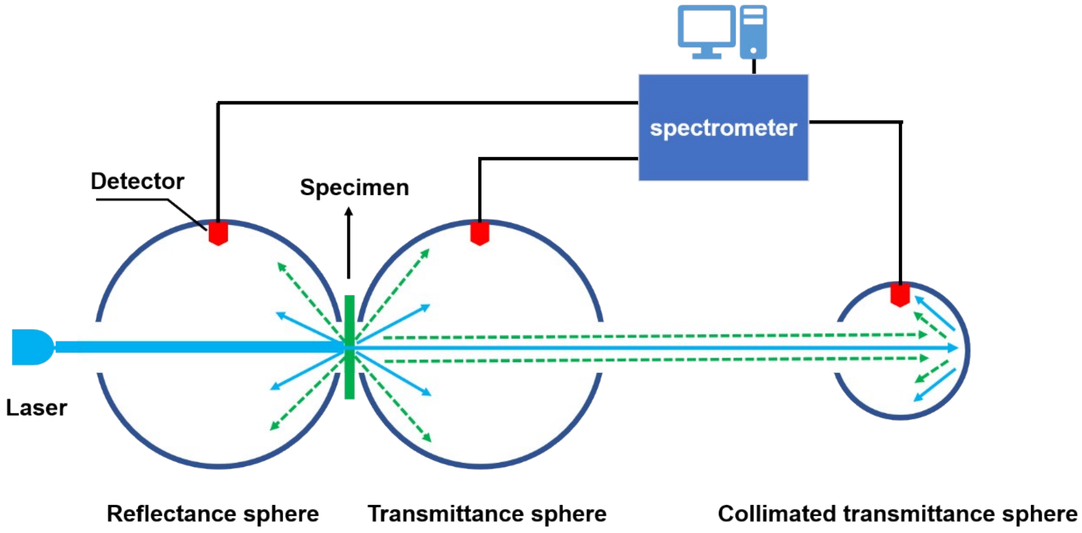

Fig. S5 Schematic of double integrating sphere system for the measurement of scattering properties for QD films.

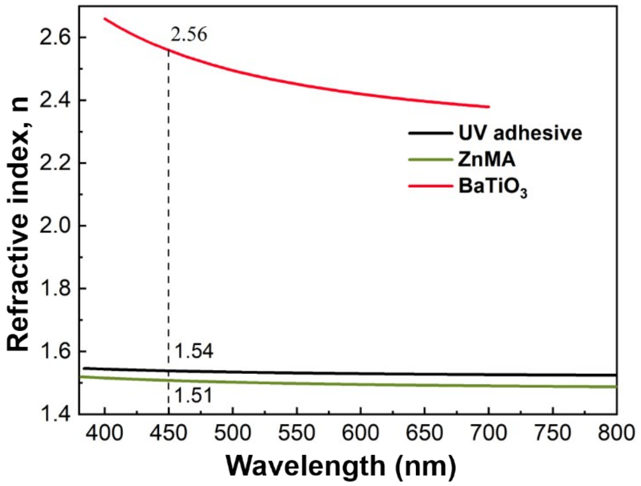

Fig. S6 Refractive indices of UV adhesive, ZnMA and $BaTiO_3$ nanoparticles.[1]

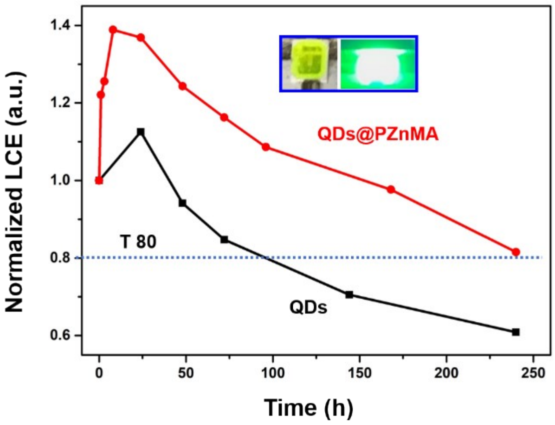

Fig.S7 Normalized decay of light conversion efficiency (LCE) of green light green emitting QDs and QDs@PZnMA on blue emitting GaN LED chips (808 mW/cm^2, 20 mA @ 3V) measured in 70 % RH in air for continuous working times. The dashed line indicated that the lifetime for the samples was reduced to 80 % (T80).

Table S1. List of measured optical results of QD samples before and after coating with PZnMA (E_X=450 nm).

Samples	QY (%)	Peak (nm)	FWHM (nm)
QDs (In solvent)	82.0	516	41
QDs (Thin film)	55.2	541	37
QDs@PZnMA (Thin film)	54.1	536	41

1 S. H. Wemple, M. Didomenico and I. Camlibel, *J. Phys. Chem. Solids*, 1968, **29**, 1797.
